# Development of Real-Time Dual-Display Handheld and Bench-Top Hybrid-Mode SD-OCTs

**DOI:** 10.3390/s140202171

**Published:** 2014-01-27

**Authors:** Nam Hyun Cho, Kibeom Park, Ruchire Eranga Wijesinghe, Yong Seung Shin, Woonggyu Jung, Jeehyun Kim

**Affiliations:** 1 School of Electrical Engineering, Kyungpook National University, 1370, Sankyuk-dong, Buk-gu, Daegu 702-701, Korea; E-Mails: nhcho@knu.ac.kr (N.H.C.); pepl10@naver.com (K.P.); dinushawij@gmail.com (R.E.W.); clemence634@naver.com (Y.S.S.); 2 School of Nano-Bioscience and Chemical Engineering, Ulsan National Institute of Science and Technology 100, Banyeon-ri, Eonyang-eup, Ulju-gun, Ulsan 689-798, Korea

**Keywords:** OCT, handheld probe, GPU, real time, dual display

## Abstract

Development of a dual-display handheld optical coherence tomography (OCT) system for retina and optic-nerve-head diagnosis beyond the volunteer motion constraints is reported. The developed system is portable and easily movable, containing the compact portable OCT system that includes the handheld probe and computer. Eye posterior chambers were diagnosed using the handheld probe, and the probe could be fixed to the bench-top cradle depending on the volunteers' physical condition. The images obtained using this handheld probe were displayed in real time on the computer monitor and on a small secondary built-in monitor; the displayed images were saved using the handheld probe's built-in button. Large-scale signal-processing procedures such as k-domain linearization, fast Fourier transform (FFT), and log-scaling signal processing can be rapidly applied using graphics-processing-unit (GPU) accelerated processing rather than central-processing-unit (CPU) processing. The Labview-based system resolution is 1,024 × 512 pixels, and the frame rate is 56 frames/s, useful for real-time display. The 3D images of the posterior chambers including the retina, optic-nerve head, blood vessels, and optic nerve were composed using real-time displayed images with 500 × 500 × 500 pixel resolution. A handheld and bench-top hybrid mode with a dual-display handheld OCT was developed to overcome the drawbacks of the conventional method.

## 1. Introduction

OCT is an imaging technology that is based on a low-coherence-length light source [1]. This technology can provide non-invasive, high-resolution, real-time imaging [2,3]. OCT systems have been extensively used for imaging in the fields of ophthalmology [4–7], dermatology [8], and dentistry [9–11]. Another advantage of these systems is the possible miniaturization of OCT beam-delivery systems by utilizing an optical fiber and micro-optics; this has been used in endoscopes and catheters [12,13], microscopes [14], needle probes[15,16], and handheld scanners [17–19]. These OCT characteristics are well suited for diagnostic imaging in scenarios of limited time, space, and subject motion [20].

In this paper, we developed a dual-monitor handheld OCT probe, a high-resolution compact portable OCT main system, and a real-time GPU signal-processing base, which is user-friendly and can be conveniently used on volunteers as an ophthalmologic diagnostic system. This system offers accurate and informative 2D-based reconstructed 3D volume images, providing more clinical information compared with the conventional methods, which makes it more convenient for the physicians' and patients' use. Images of the retina and optic-nerve head were displayed by using the recordings obtained from several volunteers in order to reduce the physical constraints on the volunteers and to validate the system. A precise diagnosis with various types of accurate information can be obtained by using the developed real-time 2D-based reconstructed 3D images acquired by using a dual-display-based handheld spectral-domain-OCT (SD-OCT) system.

## 2. Experimental Section

### 2.1. Compact Portable SD-OCT System

**Figure 1.**
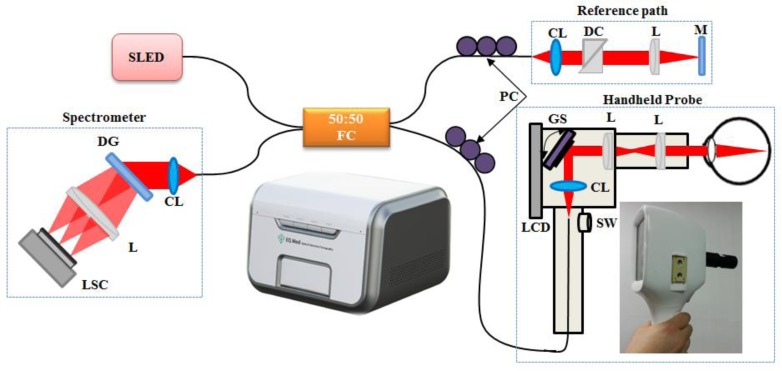
Schematic presentation and photograph of the compact portable SD-OCT system and dual-display handheld probe. Abbreviations: SLED, super-luminescent diode; FC, fiber coupler; PC, polarization controllers; CL, collimator; DC, dispersion compensation unit (prism pair); L, lens; M, mirror; DG, diffraction grating; LSC, line-scan camera; SW, switch; LCD, liquid-crystal display; GS, galvanometer scanner.

For simplicity and easier manipulation, we divided our system into five main parts: the OCT main system, the handheld probe, the bench-top-type cradle computer, and the computer monitor. The main OCT system is composed of a compact portable system (dimensions: 420-mm width × 340-mm depth × 300-mm height) that contains the optical setup for a spectrometer, reference path, and built-in light source. Additional controllers and power supplies were included in the system as well. A schematic presentation and photograph of the developed SD-OCT system and handheld probe is shown in Figure 1. A 12-bit complementary-metal–oxide–semiconductor (CMOS) line-scan camera (AVIIVA EM4 2,048 pixels, E2V, Tarrytown, NY, USA) with an effective line rate of 70,000 lines/s in 2,048-pixel mode was used as the detector of the SD-OCT system. A focusing lens with a lens diameter of 2 inches (AC508-075-B, f = 75 mm, Thorlabs, Newton, NJ, USA) was used to focus the light on the cell of the line-scan camera. An achromatic doublet lens was used to minimize the aberration and dispersion of the lenses. A transmission-type diffraction grating (spatial frequency: 1,800 lpmm (lines per millimeter), nominal angle of diffraction (AOD): 46.05°, Wasatch Photonics, Logan, UT, USA) was adapted to enhance the light efficiency in the detection path. A fiber-based Michelson interferometer was implemented with a super-luminescent diode junction (λo = 850 nm, Δλ = 55 nm, Exalos Ltd., Schlieren, Switzerland) as a light source. The light source was split into sample and reference paths, terminated by a stationary mirror. The reference path for retinal imaging also contained a dispersion compensation unit (prism pair) to account for the dispersion within the optics of the human eye and the sample path. In the sample path, a versatile handheld probe was implemented with a 2-m-long optical fiber, galvanometer scanner control (GVS002, Thorlabs), switch-controlled electrical wire, and liquid-crystal-display (LCD) monitor-controlled Universal Serial Bus (USB).

A probe at the end of the sample path delivered light to the sample and collected back-scattered light from different depths in the sample. B-mode scanning was performed using a galvanometer scanner at the back focal plane of the objective lens at the handheld probe. The B-mode maximum scan range was 3 mm. By using a mirror in the sample path, the imaging depth was found to be 3.5 mm in air. The system sensitivity was approximately 82 dB near zero optical delay when the camera was set at an exposure time of 14.1 μs. The theoretical sensitivity was approximately 96 dB because the ideal efficiency of the spectrometer was 73%, and the power at the sample path was 1.8 mW. The sensitivity of the developed system was lower than the theoretical value, mainly because of the insertion loss (−10.6 dB) between the fiber optics and the 2-D galvanometer scanner in the sample path. Losses in the other optical parts further reduced the sensitivity (−3.4 dB). The developed system had axial and lateral resolutions of 6 μm and 15 μm, respectively. The detected OCT signals were transferred to a host memory in the computer mounted with four CPUs (Core 2 Quad Q8200, 2.33-GHz clock rate, Intel, Santa Clara, CA, USA) through a frame grabber (PCIe-1433, 850-MB/s bandwidth, National Instruments, Austin, TX, USA) bandwidth over two camera-link cables. The galvanometer scanner was driven by the computer with a data acquisition board (PCIe-6321, National Instruments) that provided two analog outputs. The computer also contained a graphics card (GeForce GTX 480, 700-MHz clock rate, 480 CUDA processors, NVIDIA, Santa Clara, CA, USA).

### 2.2. GPU-Based OCT Software System and Performance

**Figure 2.**
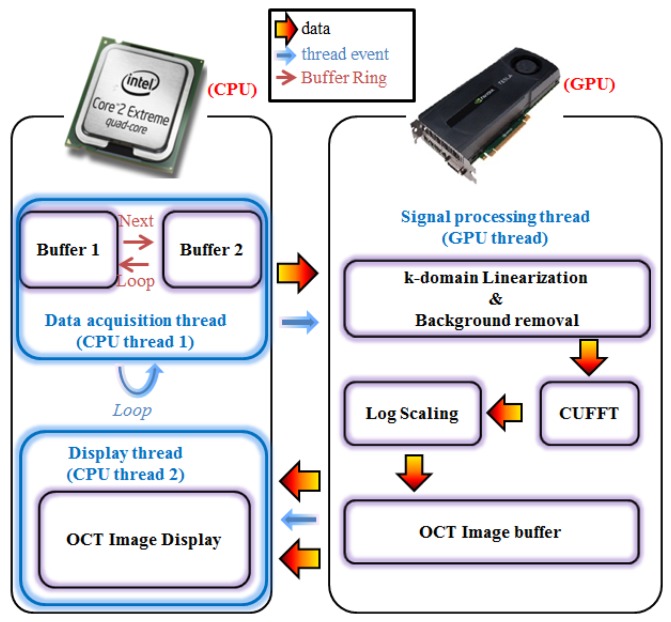
The architecture of the SD-OCT system with signal-processing part implemented in a GPU.

The software that controlled the SD-OCT system was programmed using Compute Unified Device Architecture (CUDA) version 3.2 for Labview and a GPU program for the dynamic-link-library (DLL) project of the Visual Studio 2008 programming suite. Figure 2 shows the block diagram of the SD-OCT program, including the flow of the data path, the thread events, and the buffer ring. First, the data acquisition thread stores the incoming two-dimensional signals into the first buffer allocated in the host memory and calls a signal-processing thread. Next, the self-iterated acquisition thread continuously transfers the incoming signals to the second buffer without any temporal delay between the acquisition events. The signal-processing thread copies the frame data stored in the buffers of the host memory (computer) through the peripheral component interconnect express (PCIe) ×16 2.0 interface into the device's memory (GPU). Later, the processing divides 480 CUDA sub-processors to further process the signal for OCT. We adopted the GPU to provide fast image processing and display [21]. With GPU processing, we could implement the real-time display feature after performing massive data processing, including full-range k-domain linearization [22]; background noise removal [21,23]; FFT; and log-scaling processes [24]. The reconstructed OCT images are transferred back to the host memory to be displayed in real time. The speed of the system is 56 frames/s in the SD-OCT system with an image size of 1,024 × 512 pixels.

### 2.3. Comparison of a Commercial OCT and an OCT Probe

**Figure 3.**
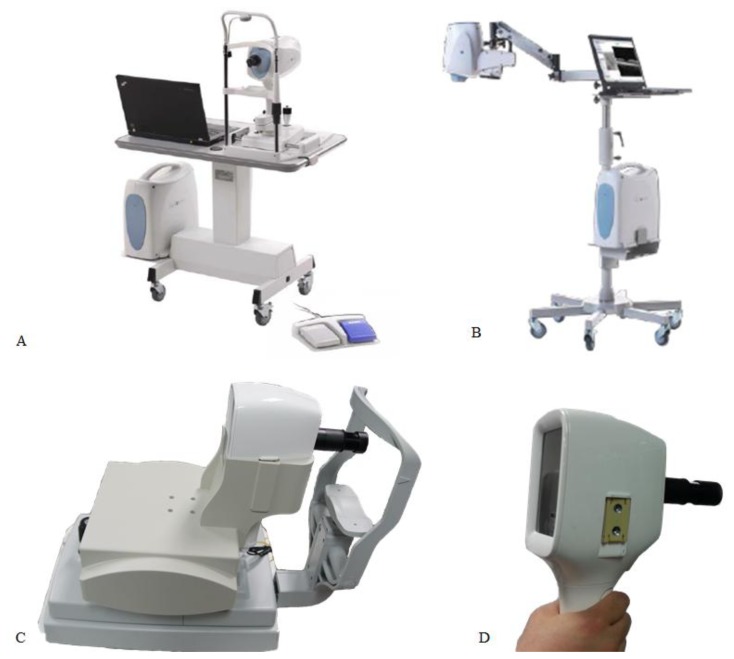
Comparison of a commercial portable OCT and handheld and bench-top hybrid OCT probes: (**a**) commercial spectral-domain OCT for all clinical applications; (**b**) commercial spectral-domain OCT for post-op patients; (**c**) bench-top handheld probe for volunteers in normal physical condition; and (**d**) handheld probe for volunteers in abnormal physical condition.

Figure 3a,b shows the recently developed portable commercial OCT system (ivue^®^, Optovue Inc., Fremont, CA, USA) [25–27]. Figure 3a shows the OCT system developed for use in all clinical applications, whereas Figure 3b shows the OCT system developed for use in surgical applications. The newly developed commercial OCT probe allows the imaging to be performed for various body positions. However, this probe is not suitable for some scenarios such as the imaging of two different body positions when it becomes necessary to use an additional arm for probe mounting. In addition, the probe is not convenient for OCT imaging of infants and children. Finally, the user may find it inconvenient to hold the probe by hand while being OCT imaged owing to the excessive probe size. Therefore, we developed a new compact portable-type OCT system that can be used as a handheld-probe-type system and also as a bench-top-type system for image acquisition according to the volunteer's condition. Figure 3c shows the SD-OCT handheld probe mounted on the bench-top cradle. Physically steady volunteers can be diagnosed by using the handheld probe, and the image can be analyzed by using the built-in LCD monitor (121 mm wide and 80 mm high). In addition, the images can be easily saved by using the joystick, which makes the physician's task more convenient while examining the patient. Figure 3d shows the handheld probe developed for uncomfortable volunteers. The handheld probe mainly consists of a handheld-probe main body, handgrip, handheld-probe adapter and a thin-film-transistor (TFT) LCD. The dimensions of the handheld probe can be shown as (main body: 90-mm width × 95-mm depth × 130-mm height; handgrip: 120 mm; adapter: 100 mm), and its weight is about 826 g. First, the system was assembled after improving the sensitivity by optically aligning the system by using optical simulation software (Zemax, Redmond, WA, USA) and after designing electronic components by using 3D CAD (Solid Works, Waltham, MA, USA) software. The locations of the optical and electrical components were determined, and these components were carefully positioned to bear any friction and external factors in order to minimize vibration. Therefore, the handheld probe consisted of various optical elements such as a collimator, galvanometer scanner, and scan lens (AC254-050-B, f = 50 mm, Thorlabs). Second, the diagnosis could be performed by comfortably holding the handgrip of the handheld probe. This handgrip facilitates protection by covering the save switch, optical fiber, and electrical wires. Third, the probe adapter was composed by using a circular tube, as the posterior chamber imaging ocular lens (AC254-030-B, f = 30 mm, Thorlabs) could be fixed inside the circular tube. The volunteer's eye can be kept in contact with the extreme edge of the probe adapter, which is located at the focal distance of the ocular lens. Motion artifacts in the image can be minimized, and a high OCT resolution can be achieved by using the probe adapter. The retinal focal length is different for different individuals. Thus, the developed system is convenient for retinal imaging because the adapter can be adjusted according to the calculated focal distance of a volunteer. Finally, by considering a high resolution, a 4.3-inch TFT-LCD with a resolution of 480 × 272 pixels was specially fixed as a secondary monitor to the handheld probe for image display. The secondary monitor was composed by considering the shape of the probe, which can obtain a 16:9 widescreen display ratio. Images were displayed vertically and horizontally according to the condition of the volunteer, and high-resolution images were obtained. The interface was implemented by using USB 2.0 for the computer and data communication.

## 3. Results

**Figure 4.**
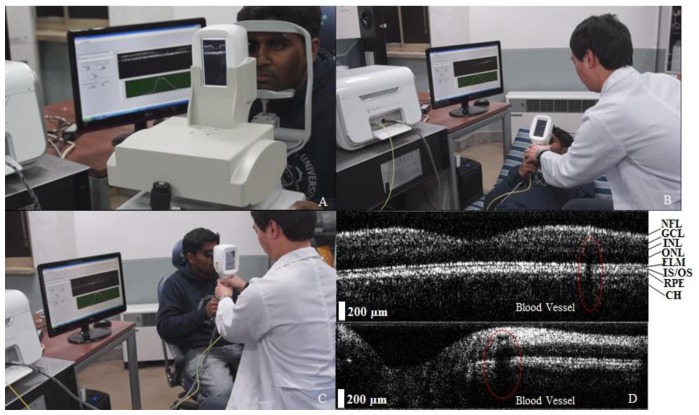
Photographs of the real-time dual-display handheld imaging probe and 2D images of a retina or an optic disc. (**a**) Bench-top mode (Media 1); (**b**) Handheld mode 1 (Media 2); (**c**) Handheld mode 2 (Media 3); (**d**) Retina and optic-nerve head of 2D OCT images. Abbreviations: NFL, nerve fiber layer; GCL, ganglion cell layer; INL, inner nuclear layer; ONL, outer nuclear layer; ELM, external limiting membrane; IS/OS, junction between the inner and outer segments of the photoreceptors; RPE, retinal pigment epithelium; CH, choroid.

To test the developed system, volunteers were examined according to their various physical conditions. Simulation imaging was taken for the normal human eye retina as a pre-experiment. Figure 4a shows a clearly observed image of the retina, blood vessels, and optic-nerve head, which can be viewed simultaneously on the computer monitor and on the handheld probe dual monitor when a physically steady volunteer is examined during the experiment. Figure 4b shows the OCT imaging method applied to a physically unsteady volunteer who was examined in this experiment. The focal lengths of the lenses were optimized for the retinal image that was displayed when the volunteer's eye was kept in contact with the extreme bottom edge of the probe adapter. The retinal image at the handheld probe is likely to be sharper than the bench-top OCT image because the handheld probe can be moved freely to obtain an informative image. Figure 4c shows the OCT imaging performed by using the handheld probe. The image acquisition was performed in a sitting position for infants and children who were unable to keep their faces on the bench-top cradle. It can be confirmed that rapid, high-resolution, and convenient imaging was performed by using the secondary monitor, rather than by using the computer monitor. Figure 4d shows the OCT images of an *in vivo* human retina and optic nerve obtained by using the handheld probe. It can be confirmed that eight layers of the retina and blood vessels can be observed clearly. In particular, the ELM, IS/OS, and RPE layers, which are among the important layers in the field of ophthalmology, can be viewed as well. In addition, a clear OCT image can be acquired for multiple layers of the optic nerve, along with blood vessels.

**Figure 5.**
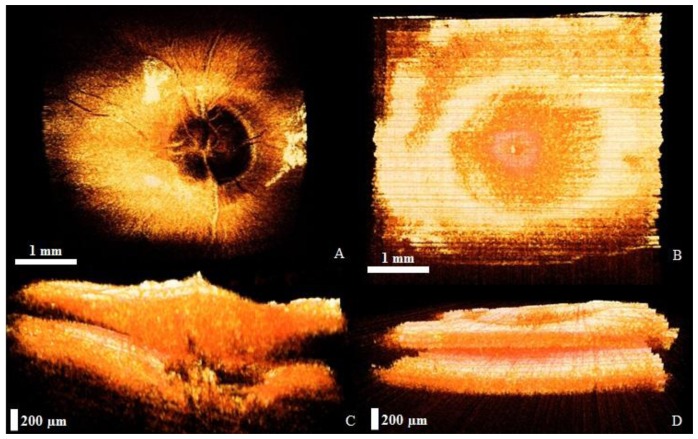
3D images of the human posterior chamber. (**a**) Optic-nerve head projection view (movie 4); (**b**) Retinal projection view (movie 5); (**c**) Optic-nerve head side view (movie 6); (**d**) Retinal side view (movie 7).

Figure 5 shows the OCT images of optic-nerve head and retina, which are among the most important fulcrums of the human eye posterior chamber, because most eye diseases are generated in these eye fulcrums. The 3D OCT images of the optic nerve and retina were reconfigured using real-time-imaged 2D OCT images. For 3D imaging, sequential 2D images (B-mode) were continuously saved and displayed in real-time during scanning. The dimensions of the acquired 3D volume were 3 × mm × 3 mm × 500 μm with 2,048 axial sampled points in depth along each A-scan and with each cross-sectional B-mode composed of 1,000 A-scans or columns of data. The dimensions of the reconfigured OCT image are 500 × 500 × 500. Figure 5a,b shows the reconfigured 3D projection view of the optic-nerve head and retina. The 3D image confirms that the optic nerve and blood vessels in all layers are well-connected to each other. Figure 5c,d shows the reconfigured 3D OCT images of the optic-nerve head and retina that were acquired by using 3D side-view images connected to 2D images. The OCT images confirm that no layer has vibrations and that the optic nerve and blood vessels in all layers are well-connected to each other.

## 4. Conclusions

In this paper, a bench-top and handheld hybrid mode SD-OCT was developed on behalf of infants, children, and volunteers who are unable to move comfortably owing to various physical conditions. The proposed system is a specially developed compact portable main system that can be moved easily. The posterior chamber of a normal human eye can be imaged using a handheld probe fixed to the bench-top cradle. The handheld probe was constructed to obtain the OCT images of the volunteers who were elderly and had physical disabilities. The main OCT system was developed as a compact portable-type system that contained an optical spectrometer, a reference path, and a light source, as mentioned above. Additional controllers and power supplies were included in the system. The bench-top handheld-probe OCT system was designed for the volunteers who had no physical disabilities and were able to move freely. The handheld probe OCT system was designed on behalf of the infants, children, and aged volunteers who had physical disabilities. In this case, it was not necessary to view the OCT image on the computer monitor, as the physician could view it on the built-in second monitor in the handheld probe and save the image while diagnosing the volunteer at the same time. The developed OCT system acquired images by varying the length of the probe adapter that varied according to the length of the volunteer's posterior chamber. Motion artifacts in the image could be minimized if the eye was kept in contact with the adapter. The developed OCT system consists of an appropriate GPU for signal processing that can be utilized to display OCT images in real time. Various volunteers such as physically steady volunteers as well as physically unsteady volunteers, infants, and children were used as experimental subjects to obtain 2D real-time images of the retina and optic-nerve head in order to test the developed system. The 3D images that were reconstructed by using the acquired 2D images convincingly show that the optic nerve and blood vessels are well-connected to each other in all layers of the optic-nerve head and retina.

We expect that the developed bench-top and handheld hybrid mode SD-OCT system can be used for image acquisition of volunteers without considering their age or physical condition. It is necessary to perform additional clinical experiments and collect more data from various volunteers to overcome the drawbacks of the conventional method and to improve the functioning status of the developed system. In the future, three issues will need to be addressed: (1) improving the image display rate by using dual GPU techniques [28]; (2) adding a fundus camera mounted on the handheld probe; and (3) improving the OCT image resolution by using eye-tracking techniques [29]. Retinal tracking techniques have specific advantages such as removing the motion error and frame averaging to enhance contrast and remove image speckle in high-resolution images. This technique can be suitably used in a motion-sensitive handheld probe to compensate for this drawback.
